# Epidemiological and Clinical Characteristics of Cases During the Early Phase of COVID-19 Pandemic: A Systematic Review and Meta-Analysis

**DOI:** 10.3389/fmed.2020.00295

**Published:** 2020-06-11

**Authors:** Jiayun Koh, Shimoni Urvish Shah, Pearleen Ee Yong Chua, Hao Gui, Junxiong Pang

**Affiliations:** ^1^Saw Swee Hock School of Public Health, National University of Singapore and National University Health System, Singapore, Singapore; ^2^Centre for Infectious Disease Epidemiology and Research, National University of Singapore, Singapore, Singapore

**Keywords:** coronavirus, epidemiology, clinical features, systematic review, early pandemic phase, COVID-19

## Abstract

**Background:** On 29th December 2019, a cluster of cases displaying the symptoms of a “pneumonia of unknown cause” was identified in Wuhan, Hubei province of China. This systematic review and meta-analysis aims to review the epidemiological and clinical characteristics of COVID-19 cases in the early phase of the COVID-19 pandemic.

**Methods:** The search strategy involved peer-reviewed studies published between 1st January and 11th February 2020 in Pubmed, Google scholar and China Knowledge Resource Integrated database. Publications identified were screened for their title and abstracts according to the eligibility criteria, and further shortlisted by full-text screening. Three independent reviewers extracted data from these studies, and studies were assessed for potential risk of bias. Studies comprising non-overlapping patient populations, were included for qualitative and quantitative synthesis of results. Pooled prevalence with 95% confidence intervals were calculated for patient characteristics.

**Results:** A total of 29 publications were selected after full-text review. This comprised of 18 case reports, three case series and eight cross-sectional studies on patients admitted from mid-December of 2019 to early February of 2020. A total of 533 adult patients with pooled median age of 56 (95% CI: 49–57) and a pooled prevalence of male of 60% (95% CI: 52–68%) were admitted to hospital at a pooled median of 7 days (95% CI: 7–7) post-onset of symptoms. The most common symptoms at admission were fever, cough and fatigue, with a pooled prevalence of 90% (95% CI: 81–97%), 58% (95% CI: 47–68%), and 50% (95% CI: 29–71%), respectively. Myalgia, shortness of breath, headache, diarrhea and sore throat were less common with pooled prevalence of 27% (95% CI: 20–36%), 25% (95% CI: 15–35%), 10% (95% CI: 7–13%), 8% (95% CI: 5–13%), and 7% (95% CI: 1–15%), respectively. ICU patients had a higher proportion of shortness of breath at presentation, as well as pre-existing hypertension, cardiovascular disease and COPD, compared to non-ICU patients in 2 studies (*n* = 179).

**Conclusion:** This study highlights the key epidemiological and clinical features of COVID-19 cases during the early phase of the COVID-19 pandemic.

## Introduction

On 29th December 2019, a cluster of cases displaying the symptoms of a “pneumonia of unknown cause” was identified in Wuhan, Hubei province, China (1). Further investigations found that these cases were linked to Huanan Seafood Wholesale Market. The Wuhan pneumonia cluster rapidly spread across the globe with initial reports of cases in Thailand, Japan and Korea (2). The World Health Organization (WHO) subsequently declared COVID-19 (then named 2019-nCoV) outbreak a Public Health Emergency of International Concern (PHEIC) on 30th January 2020. By then, there were 7,818 COVID-19 cases reported worldwide, with 7,736 cases from China and 82 cases from 18 other countries (3).

The Novel Coronavirus Research Team in China identified and characterized the causal pathogen, which was named severe acute respiratory syndrome coronavirus-2 (SARS-CoV-2) (4). Studies have shown that the novel pathogen bears similarity to two other global threats, SARS-CoV and Middle East respiratory syndrome coronavirus (MERS-CoV), as it belongs to the same family of viruses (4, 5). SARS-CoV-2 shares 79% sequence identity with SARS-CoV and 50% with MERS-CoV (6). This resemblance has key implications on how COVID-19 manifests in affected individuals, and experience with MERS and SARS can help guide researchers and authorities in tackling COVID-19 (7).

With the progression of the outbreak into a pandemic, health authorities have realized that community transmission of COVID-19 is becoming more difficult to avoid (8). Instead the focus has been to ensure that health systems are able to cope with COVID-19 hospitalizations, and that vulnerable populations prone to the severe effects of COVID-19 receive appropriate supportive care (9).

Even as numbers have dropped in China while other regions like Europe have become new epicenters of the pandemic (10), there is still limited knowledge on the risk factors and severity of COVID-19 (11). This systematic review primarily aims to review the epidemiological and clinical characteristics of cases admitted to hospitals for COVID-19 at the early phase of the pandemic. Moreover, this review will examine the potential differences between cases who were admitted to ICU and those who weren't.

## Methods

### Search Strategy

**Figure 1:**
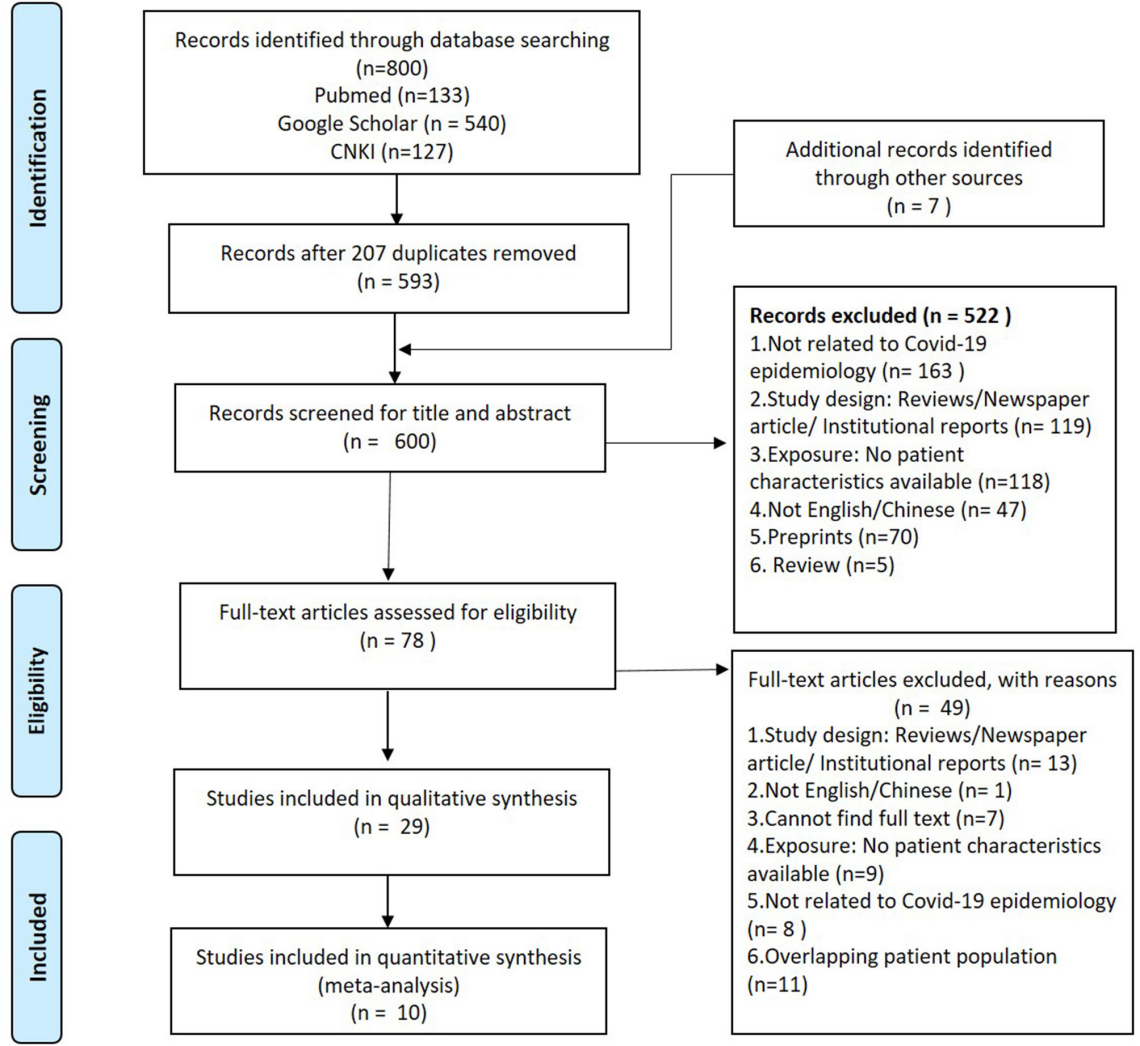
PRISMA flow diagram of the search strategy for peer-reviewed studies up till 11th February 2020.

A systematic search was conducted with three databases—Pubmed, Google Scholar and China Knowledge Resource Integrated (CNKI) database according to the Preferred Reporting Items for Systematic Reviews and Meta-Analyses (PRISMA) guidelines (Figure 1) and checklist (Figure S1). Keywords, such as “2019-nCoV,” “2019 novel coronavirus,” “nCoV,” “新型冠状病毒,” “新型肺炎,” and “Wuhan pneumonia” were used in the search to identify articles published on or before 11th February, 2020 in English or Chinese. The cut-off date was aimed to coincide with the announcement of COVID-19 as a PHEIC by the WHO and also the early phase of the pandemic. This date coincided with the official naming of disease as COVID-19, and hence this term was not included in the search. The publications were imported and managed in EndnoteX9. Inclusion criteria for the studies was based on the PICOS framework (Table S1). The studies excluded in this review were preprints, editorials, news articles or reviews of selected articles. We included brief reports and correspondences for this systematic review.

### Data Extraction

Three reviewers independently extracted the relevant data from eligible studies and any disagreement in the extraction was resolved by a fourth reviewer. The data were extracted to an excel sheet template which included information on the study details (type of article, study type, etc.), patient demographics (age, gender, exposure, etc.), symptoms, chest imaging, clinical management (treatment, respiratory support) and clinical outcomes. Aggregate patient data, and available data stratified into ICU and non-ICU, were recorded as separate rows.

### Quality Control

Each selected paper was assessed with Murad et al.'s Methodology Assessment tool for case series and case reports, which was based on four domains—selection, ascertainment, causality, and reporting (12). The results from this assessment tool signaled the quality of case series/reports for qualitative and quantitative synthesis. Risk of bias was summarized and visualized using Revman5 (Figure S2).

### Data Analysis

The frequencies and proportions of patients' characteristics were reviewed. Logit and double arcsine transformation methods were used in proportional meta-analysis. The pooled prevalence of demographic factors, clinical characteristics and outcomes were calculated with 95% confidence intervals, and forest plots generated using R statistical software version 3.6.3. A random-effects model was used, which is a more conservative approach, considering the variability of epidemiological and clinical characteristics. Only studies with acceptable risk of bias, and adult populations were included in the meta-analysis.

## Results

### Literature Search Results and Selected Study Characteristics

A total of 800 studies were obtained from search results and 593 were reviewed after excluding 207 duplicates. An additional seven studies were found from other sources. The title and abstracts of 600 studies were screened according to the eligibility criteria. Five hundered twenty-two studies did not meet the eligibility criteria and were excluded (Figure 1), and 78 articles were shortlisted for full text screening. After reviewing the full text, a total of 29 publications were included in the systematic review.

Among the 29 eligible studies selected, a total of 578 COVID-19 cases were reported. Of these, 23 patients were reported from 18 case reports (Table S2). The case reports described patients from China (4, 13–19), Vietnam (20), Germany (21), USA (22), South Korea (23), and Nepal (24). The remaining 533 adults and 22 children cases were detailed in three case series and eight cross-sectional studies that were all from China (Table S2); Five studies (25–29) were from the city of Wuhan, while the rest of the studies were from other parts of China (25, 26, 30–34). Although two studies reported patient data from Wuhan Jin Yin-tan hospital, admission dates were not overlapping; patients in Huang et al.'s study was admitted from 16-Dec-19 to 02-Jan-20 while patients from Chen et al.'study were admitted from 01-Jan-20 till 20-Jan-20.

### Risk of Bias

All 11 case series had acceptable risk of bias (unclear or high risk of bias in ≤1 domain). Reasons for potential bias included using secondary data from government sources, and not specifying the cut-off date for data reporting (Figure S2). Seven of the 18 case reports were of unacceptable risk of bias, mostly due to lack of explanation of how patients were selected, and unstandardized reporting of patient variables amongst the cases.

### Epidemiological Characteristics

Across the 11 case series and cross-sectional studies selected from full-text review, there were different proportions of case severities—seven studies consisted entirely of COVID-19 pneumonia cases (*n* = 482) (25–28, 30, 33, 35). On the other hand, Wang et al. drew data from National Health Commission sources, and reported data exclusively on the first 17 COVID-19 deaths across China (median age 75 and IQR: 66–82; 76% male) (34). Ten studies (*n* = 533) were on adult populations with a pooled median age of 56 (IQR:49–57). Only one study 马慧静/Ma et al. looked at pediatric patients (*n* = 22) with ages ranging from 2 months to 14 years (Table 1) (29). In five studies with patients from hospitals outside of Wuhan-−87% (95% CI: 65–100%) of the cases were either from Wuhan or had a travel history to Wuhan. Of all four studies (*n* = 415) with adult patients from Hubei province, 24% (95% CI: 1–61%) were exposed to the Hunan seafood market. The pooled median time from symptom onset to admission was 7 days (95% CI: 7–7) (Table 1). There was very limited reporting of epidemiological information on the other potential sources of infection, such as household or occupational risk of transmission.

**Table 1:** Patient demographic and epidemiological information for selected studies.

**City/Country Hospital**	***N* (% with pneumonia)**	**Age in years**	**Male (%)**	**Epidemiological link with Wuhan (%)**	**Hunan seafood market (%)**	**Duration from symptoms onset to medical event**	**Reference**
**CROSS-SECTIONAL STUDIES AND CASE SERIES**							
Shenzhen/China The University of Hong Kong, Shenzhen Hospital	6 (100%)	Median 50 (IQR: 36–56)	3 (50%)	6 (100%)	–	–	Chan et al. (30)
Beijing/China 1. Beijing Tsinghua Changgung Hospital 2. Beijing Anzhen Hospital 3. Chinese PLA General Hospital	13 (62%)	Median 34 (IQR: 34–48)	10 (77%)	12 (92%)	–	To hospitalization–mean 1.6 days	Chang et al. (31)
Wuhan/China Wuhan Jin Yin-tan hospital	99 (100%)	Median 55.5 (SD, range: 13.1, 21–82)	67 (68%)	*Not applicable (Wuhan hospital)*	49 (49%)	–	Chen et al. (27)
Qingdao, Zhuhai, and Nanchang/China 1. The First Affiliated Hospital of Nanchang University 2. The Affiliated Hospital of Qingdao University 3. The Fifth Affiliated Hospital, Sun Yat-sen University	21 (85%)	Mean 51 (SD, range: 14, 29–77)	13 (62%)	17 (81%)	–	–	Chung et al. (32)
Wuhan/China Wuhan Jin Yin-tan hospital	41 (100%)	Median 49 (IQR 41–58)	30 (73%)	*Not applicable (Wuhan hospital)*	27 (66%)	To first hospital admission–median 7 days (IQR: 4–8)	Huang et al. (25)
Wuhan, Shiyan, Jingzhou, Yichang, Xiaogan/China (Hubei) 1. Tongji Hospital 2. Central Hospital of Wuhan 3. Taihe Hospital 4. Jingzhou Central Hospital 5. The First People's Hospital of Jingzhou 6. The People's Hospital of Zhou 7. The Central Hospital of Xiaogan 8. The Sixth Hospital of Wuhan 9. Central Hospital of Enshi Tujia	137 (100%)	Median 57 (Range: 20–83)	61 (45%)	–	0	To dyspnea or significant symptoms–median 7 days (range 1–20 days)	Kui et al. (26)
Shanghai/China Shanghai Public Health Clinical Centre	51 (100%)	Mean 49 (SD: 16)	25 (49%)	50 (98%)	–	To ICU admission–median 9·5 days (IQR: 7–12·5)	Song et al. (33)
Wuhan/China Zhongnan Hospital of Wuhan University	138 (100%)	Median 56 (IQR: 42–68)	75 (55%)	*Not applicable (Wuhan hospital)*	12 (8.7)	To hospital admission–median 7 days (IQR:4–8) To dyspnea−5 days (1–10)	Wang et al. (28)
Across China From National Health Commission Website	17 (Unspecified)	Median 75 (Range: 48–89)	13 (76%)	–	–	To death–median 14 days (range, 6–41)	Wang et al. (34)
Shanghai/China 1. Shanghai Jiao Tong Affiliated Sixth People's Hospital 2. Jinshan Branch Hospital	10 (100%)	Range 24–65	5 (50%)	8 (80%)	–	–	杨涛/Yang et al. (35)
Wuhan, China^*^ Wuhan Children's' Hospital	22 (86%)	Median 4 (Range: 2 months to 14 years)	12 (55%)	*Not applicable (Wuhan hospital)*	Infected person exposure: 17 (77%)	–	马慧静/Ma et al. (29)^*^
**Pooled Prevalence (95% CI)**		**Median 56^+^** **(49–57)**	**60%** **(52–68%)**	**87%** **(65–100%)**	**24%** **(1–61%)**	**Median 7 days to admission (7–7)**	
CASE REPORTS (WORLDWIDE)^**^							
Kathmandu/Nepal Kathmandu hospital	1 (Pneumonia)	32	Male	Studying in Wuhan	–	To discharge−13 days	Bastola et al. (24)
Washington/USA Providence Regional Medical Centre	1 (Pneumonia)	35	Male	Visited family in Wuhan	–	To admission−4 days To recovery−12 days	Holshue et al. (22)
Seoul/South Korea Seoul National University College of Medicine	1 (Pneumonia)	35	Female	Lived in Wuhan	–	To resolution of fever−11 days To symptoms recovery−14 days	Kim et al. (23)
Ho Chi Minh, Vietnam Cho Ray Hospital	1 (Pneumonia)	65	Male	Visited Wuhan	–	To admission−4 days To stability−13 days	Phan et al. (20)
		27	Male	Met father in Nha Trang	–	–	
Munich, Germany Medical Center of the University of Munich	1 (Pneumonia)	33	Male	Exposed by colleague from Shanghai		To recovery−4 days	Rothe et al. (21)

* *Study 马慧静/Ma et al. not included in pooled figures as subjects are children*.

** *Case reports from patients in China found in Supplementary Material*.

+ *Only studies reporting median as a summary for age are pooled*.

Of the 18 case reports (Table S3), the age range of 23 patients reported was from 3 months to 65 years; 13 patients (56%) were male. There were 2 reported cases in literature who did not have any exposure to Wuhan or travel history to China—instead the patients were exposed to a symptomatic father and an asymptomatic colleague from Shanghai, respectively, thereby confirming local transmission in Vietnam as well as transmission from asymptomatic cases in Germany (Table 1) (20, 21).

### Comorbidities

Out of 13 case reports (*N* = 16) that documented details of chronic conditions, 9 (56%) did not have any comorbidities (Table S3). The most common comorbidities found were diabetes, hypertension and cardiovascular disease (CVD). In pooled analyses of at least 400 patients in the 10 studies with acceptable risk of bias, the most prevalent comorbidities were hypertension (17%, 95% CI: 7–28%), diabetes (10%, 95% CI: 6–15%), and cardiovascular disease (12%, 95% CI: 3–23%) (Table 2 and Figure S3). In 295 subjects with available data, 45% (95% CI: 37–56%) of patients were found to have any co-morbidity.

**Table 2:** Comorbidities in patients from selected studies.

**Study (References)**	***N* (% with pneumonia)**	**Diabetes**	**Hypertension**	**Cardiovascular disease**	**Malignancy**	**Chronic liver disease**	**COPD**	**Chronic kidney disease**	**Others (*n*, %)**	**Any co-morbidity**
**CROSS-SECTIONAL STUDIES AND CASE SERIES**										
Chan et al. (30)	6 (100%)	1 (22%)	2 (33%)	–	1 (22%)	–	–	–	Chronic sinusitis (22%)	–
Chang et al. (31)	13 (62%)	–	–	–	–	–	–	–		–
Chen et al. (27)	99 (100%)	–	–	40 (40%)	1 (1%)	–	–	–	Digestive system disorder (11%), Endocrine system disorder (13%), Nervous system disease (1%)	50 (51%)
Chung et al. (32)	21 (85%)	–	–	–	–	–	–	–	–	–
Huang et al. (25)	41 (100%)	8 (20%)	6 (15%)	6 (15%)	1 (2%)	1 (2%)	1 (2%)	–	–	13 (32%)
Kui et al. (26)	137 (100%)	14 (10.2%)	13 (9.5%)	10 (7.3%)	2 (1.5%)	–	2 (1.5%)	–	–	–
Song et al. (33)	51 (100%)	3 (6%)	5 (10%)	1 (2%)	–	1 (2%)	1 (2%)	–	–	–
Wang et al. (28)	138 (100%)	14 (10.1%)	43 (31.2%)	20 (14.5%)	10 (7.2%)	4 (2.9%)	4 (2.9%)	4 (2.9%)	HIV (1.4%)	64 (46.4%)
Wang et al. (34)	17 (Unspecified)	5 (29%)	7 (41%)	2 (11.7%)	–	1 (6%)	1 (5.8%)	2 (11.7%)	Surgery (29.4%)	11 (64.7%)
杨涛/Yang et al. (35)	10 (100%)	0	0	0	0	0	0	1 (10%)		
马慧静/Ma et al. (29)^*^	22 (86%)	–	–	–	–	–	–	–	–	–
**Pooled prevalence (95% CI)**		**10% (6–15%)**	**17% (7–28%)**	**12% (3–23%)**	**2% (<1–5%)**	**2% (<1–4%)**	**1% (<1–3%)**	**6% (2–15%)**	**–**	**45% (34–56%)**
CASE REPORTS (WORLDWIDE)^**^										
Bastola et al. (Nepal) (24)	1 (Pneumonia)	–	–	–	–	–	–	–	–	No
Holshue et al. (USA) (22)	1 (Pneumonia)	–	–	–	–	–	–	–	–	No
Kim et al. (S. Korea) (23)	1 (Pneumonia)	Yes	–	–	–	–	–	–	–	Yes
Phan et al. (Vietnam) (20)	1 (Pneumonia)	Yes	Yes	Yes	Yes	–	–	–	–	Yes
Rothe et al. (Germany) (21)	1 (Mild infection)	–	–	–	–	–	–	–	–	No

* *Study 马慧静/Ma et al. not included in pooled figures as subjects are children*.

** *Case reports from patients in China found in Supplementary Material*.

*COPD, Chronic Obstructive Pulmonary Disease*.

Among the first 17 COVID-19 deaths in China summarized by Wang et al., there were 11 cases (64.7%) who had at least one comorbidity (Table 2) (34). Among those with intensive care unit (ICU) admission status in two studies (*n* = 179) (25, 28), patients had a greater proportion of existing comorbidities (38 and 72%, respectively) compared to non-ICU patients (29 and 37%, respectively) (Table 4). In particular, there was a higher proportion of hypertension, cardiovascular and COPD in ICU patients compared to non-ICU patients within both studies (Table 4). This difference was statistically significant only in Wang et al.'s study.

### Symptoms at Admission

**Figure 2:**
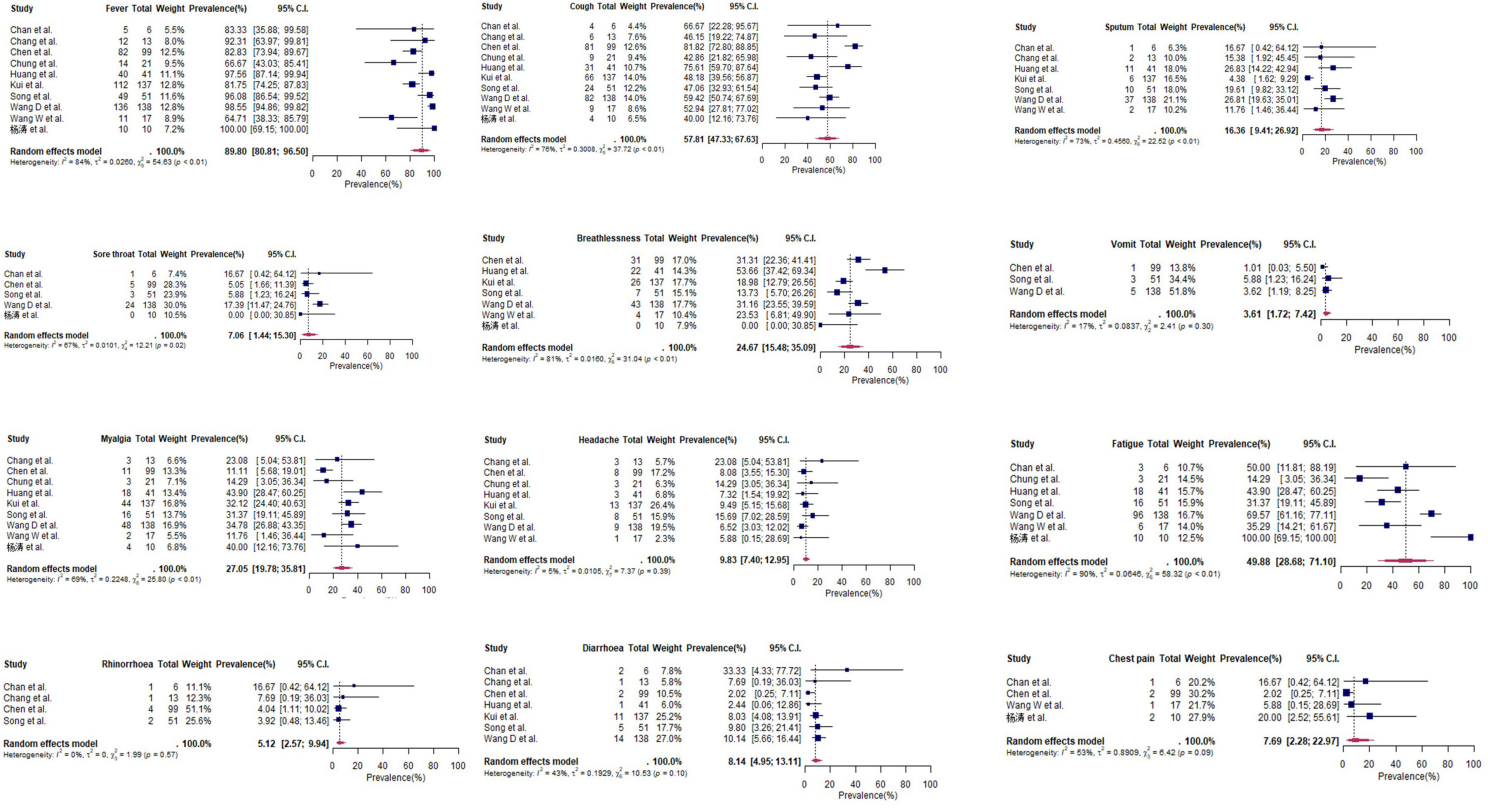
Forest plots for pooled prevalence of symptoms at admission (cross-sectional studies and case series).

For COVID-19 patients at admission (*n* = 533), symptoms with the highest pooled prevalence include fever (90%, 95% CI: 81–87%), cough (58%, 95% CI: 47–68%), and fatigue (50%, 95% CI: 29–71%) (Table 3 and Figure 2). Shortness of breath and myalgia had pooled prevalence of 25% (95% CI: 15–35%) and 27% (95% CI: 20–36%), respectively. Headache, diarrhea and sore throat showed a pooled prevalence of 10, 8, and 7%, respectively (Table 3). From case series and case reports with available information (*n* = 12), the first symptoms during onset were also fever (9 cases, 75%) and cough (4 cases, 33%) (Tables S4, S5). Amongst children in 马慧静/Ma et al.'s study (29), the prevalence of all symptoms at admission was lower compared to adult populations, except for rhinorrhea (Table 3). Within two studies, patients who required ICU admission (*n* = 179) had a significantly higher prevalence of shortness of breath (92 and 64% vs. 37 and 20%, respectively) compared to patients who did not require ICU admission (Table 4) (25, 28).

**Table 3:** Symptoms at admission presented by patients from selected studies.

**Study (References)**	***N* (% with pneumonia)**	**Fever (%)**	**Cough (%)**	**Sputum (%)**	**Sore throat (%)**	**Shortness of breath (%)**	**Vomiting (%)**	**Myalgia (%)**	**Malaise/Fatigue (%)**	**Rhinorrhoea (%)**	**Headache (%)**	**Diarrhea (%)**	**Chest pain (%)**
**CROSS-SECTIONAL STUDIES AND CASE SERIES**													
Chan et al. (30)	6 (100%)	5 (83%)	4 (67%)	1 (17%)	1 (17%)	–	–	–	3 (50%)	1 (17%)	–	2 (33%)	1 (17%)
Chang et al. (31)	13 (62%)	12 (92%)	6 (46.2%)	2 (15%)		–	–	3 (23%)	–	1 (8%)	3 (23%)	1 (8%)	–
Chen et al. (27)	99 (100%)	82 (83%)	81 (82%)	–	5 (5%)	31 (31%)	1 (1%)	11 (11%)	–	4 (4%)	8 (8%)	2 (2%)	2 (2%)
Chung et al. (32)	21 (85%)	14 (67%)	9 (43%)	–	–	–	–	3 (14%)	3 (14%)	–	3 (14%)	–	–
Huang et al. (25)	41 (100%)	40 (98%)	31 (76%)	11 (28%)	–	22 (55%)	–	18 (44%)	–	3 (8%)	1 (3%)	–	–
Kui et al. (26)	137 (100%)	112 (82%)	66 (48%)	6 (4.4%)	–	26 (19%)	–	44 (32%)	–	–	13 (10%)	11 (8%)	–
Song et al. (33)	51 (100%)	49 (96%)	24 (47%)	10 (20%)	3 (6%)	7 (14%)	3 (6%)	16 (31%)	16 (31%)	2 (4%)	8 (16%)	5 (10%)	–
Wang et al. (28)	138 (100%)	136 (99%)	82 (59%)	37 (27%)	24 (17%)	43 (31%)	5 (4%)	48 (35%)	96 (70%)	–	9 (7%)	14 (10%)	–
Wang et al. (34)	17 (Unspecified)	11 (65%)	9 (53%)	2 (12%)	–	4 (23%)	–	2 (12%)	6 (35%)	–	1 (6%)	–	1 (6%)
杨涛/Yang et al. (35)	10 (100%)	10 (100%)	4 (40%)	–	0	0	–	4 (40%)	10 (100%)	–	–	–	2 (20%)
马慧静/Ma et al. (29)^*^	22 (86%)	13 (59%)	5 (23%)	2 (9%)	1 (5%)	1 (5%)	–	–	–	3 (14%)	–	1 (5%)	–
**Pooled Prevalence** **(95% CI)**		**90%** **(81–97%)**	**58%** **(47–68%)**	**16%** **(9–27%)**	**7%** **(1–15%)**	**25%** **(15–35%)**	**4%** **(2–7%)**	**27% (20–36%)**	**50% (29–71%)**	**5%** **(3–10%)**	**10% (7–13%)**	**8%** **(5–13%)**	**8%** **(2–23%)**
CASE REPORTS (WORLDWIDE)^**^													
Bastola et al. (Nepal) (24)	1 (Pneumonia)	Yes	Yes	–	–	Yes	–	–	–	–	–	–	–
Holshue et al. (USA) (22)	1 (Pneumonia)	Yes	Yes	–		–	Yes	–	Yes	–	–	–	–
Kim et al. (S. Korea) (23)	1 (Pneumonia)	Yes	–	–	Yes	–	–	Yes	–	–	–	–	–
Phan et al. (Vietnam) (20)	1 (Pneumonia)	Yes	–	–	–	Yes	–	–	Yes	–	–	–	–
Rothe et al. (Germany) (21)	1 (Mild infection)	Yes	Yes	Yes	Yes	–	–	Yes	Yes	–	–	–	–

* *Study 马慧静/Ma et al. not included in pooled figures as subjects are children*.

** *Case reports from patients in China found in Supplementary Material*.

**Table 4:** Comorbidities and symptoms on admission, stratified by ICU admission.

**Study (References)**	**Case classification**	**Diabetes**	**Hypertension**	**Cardio-vascular disease**	**Malignancy**	**Chronic liver disease**	**COPD**	**Others (*n*, %)**	**Any co-morbidity**
**COMORBIDITIES**									
**Huang et al**. **(****25****)**									
	ICU cases (*n* = 13)	1 (8%)	2 (15%)	3 (23%)	0	0	1 (8%)	–	5 (38%)
	Non-ICU (*n* = 28)	7 (25%)	4 (14%)	3 (11%)	1 (4%)	1 (4%)	0	–	8 (29%)
*p*-value		0.17	0.93	0.32	0.49	0.68	0.14	–	0.53
**Wang et al**. **(****28****)**									
	ICU cases (*n* = 36)	8 (22.2%)	21 (58.3%)	9 (25%)	4 (11.1%)	0	3 (8.3%)	–	26 (72%)
	Non-ICU (*n* = 102)	6 (5.9%)	22 (21.6%)	11 (10.8%)	6 (5.9%)	4 (3.9%)	1 (1%)	HIV (2.2%)	38 (37%)
*p*-value		**0.009**	**<0.001**	**0.04**	0.29	0.57	0.054	–	**<0.001**
**Author**	**Case classification**	**Fever (%)**	**Cough (%)**	**Sputum (%)**	**Shortness of breath (%)**	**Myalgia (%)**	**Malaise/** **Fatigue (%)**	**Headache (%)**	**Diarrhea (%)**
**SYMPTOMS AT ADMISSION**									
**Huang et al**. **(****25****)**									
	ICU (*n* = 13)	13(100%)	11 (85%)	5(38%)	12 (92%)		7 (54%)	0	0
	Non-ICU (*n* = 28)	27 (96%)	20 (71%)	6 (23%)	10 (37%)		11 (39%)	3 (12%)	1 (4%)
*p*-value		0.68	0.35	0.32	**0.001**		0.38	0.10	0.66
**Wang et al**. **(****28****)**									
	ICU (*n* = 36)	36 (100%)	21 (58%)	8 (22%)	23 (64%)	12 (33%)	29 (81%)	3 (8%)	6 (17%)
	Non-ICU (*n* = 102)	100 (98%)	61 (60%)	29 (28%)	20 (20%)	36 (35%)	67 (66%)	6 (6%)	8 (8%)
*p*-value		>0.99	0.88	0.35	**<0.001**	0.83	0.1	0.7	0.2

*COPD, Chronic Obstructive Pulmonary Disease*.

*p-value in bold is statistically significant (<0.05)*.

### Chest Imaging at Admission

Based on chest X-ray/CT imaging results of 519 patients, bilateral involvement of lungs was shown in a high percentage of patients assessed (Table 5 and Figure S4), with a pooled prevalence of 90% (95% CI: 77–98%). Pooled analyses of studies with available data of at least 250 patients also showed that ground glass pattern was found in 59% (95% CI: 35–82%) of patients and consolidation in 31% (95% CI: 12–55%) of patients.

**Table 5:** Chest imaging at admission and treatment of patients from selected studies.

		**Chest imaging**				**Treatment**						
**References**	***N* (% with pneumonia)**	**Ground glass**	**Consolidation**	**Unilateral involvement**	**Bilateral involvement**	**Mechanical ventilation**	**High flow cannulation**	**ECMO**	**Antiviral agents**	**Renal replacement**	**Corticosteroids**	**Immunoglobulin**
**CROSS-SECTIONAL STUDIES AND CASE SERIES**												
Chan et al. (30)	6 (100%)	6 (100%)	0	–	–	–	–	–	–	–	–	–
Chang et al. (31)	13 (62%)	6 (46%)	8	–	–	–	–	–	–	–	–	–
Chen et al. (27)	99 (100%)	14 (14%)	–	25 (25%)	74 (75%)	4(4%)	13 (13%)	3 (3%)	75 (76%)	9 (%)	19 (19%)	27 (27%)
Chung et al. (32)	21 (85%)	12 (57%)	6 (29%)	–	16 (76%)	–	–	–	–	–	–	–
Huang et al. (25)	41 (100%)	–	–	–	40 (98%)	2 (5%)	10 (24%)	2 (5%)	38 (93%)	3 (7%)	9 (22%)	–
Kui et al. (26)	137 (100%)	55 (47%)	25 (21.6)	–	116 (85%)	–	119 (86.9%)	0	105 (76.6%)	–	40 (29.2%)	44 (32.1%)
Song et al. (33)	51 (100%)	39 (77%)	28 (55%)	7 (14%)	44 (86%)	–	–	–	–	–	–	
Wang et al. (28)	138 (100%)	–	–	–	138 (100%)	17 (12%)	15 (11%)	4 (2.9%)	124 (89.9)	2 (1.45%)	62 (44.9%)	–
Wang et al. (34)	17 (Unspecified)	–	–	–	–	–	–	–	–	–	–	–
杨涛/Yang et al. (35)	10 (100%)	9 (90%)	–	1 (10%)	9 (90%)	–	–	–	–	–	–	–
马慧静/Ma et al. (29)^*^	22 (86%)	6 (27%)	4 (18%)	7 (32%)	12(54%)	–	–	–	–	–	–	–
	**Pooled prevalence (95% CI)**	**59% (35–82%)**	**31% (12–55%)**	**19% (11–31%)**	**90% (77–98%)**	**7% (3–16%)**	**31% (6–77%)**	**2% (<1–5%)**	**84% (74–90%)**	**5% (2–14%)**	**29%** **(18–42%)**	**30%** **(24–36%)**
CASE REPORTS (WORLDWIDE)^**^												
Bastola et al. (Nepal) (24)	1 (Pneumonia)	–	–	Yes	–	–	–	–	–	–	–	–
Holshue et al. (USA) (22)	1 (Pneumonia)	–	–	–	–	–	Yes	–	Yes (Remdesivir)	–	–	–
Kim et al. (S. Korea) (23)	1 (Pneumonia)	Ye s	Yes	Yes	–	–	Yes	–	Yes (Lopinavir/ Ritonavir)	–	–	–
Phan et al. (Vietnam) (20)	1 (Pneumonia)	–	Yes	–	–	–	Yes	–	Yes	–	Yes	–
Rothe et al. (Germany) (21)	1 (Mild infection)	–	–	–	–	–	–	–	–	–	–	–

* *Study 马慧静/Ma et al. not included in pooled figures as subjects are children*.

** *Case reports from patients in China found in Supplementary Material*.

*ECMO, Extracorporeal Membrane Oxygenation*.

### Treatment, Complications, and Outcomes

Invasive mechanical ventilation was administered in 7% of patients in three cross-sectional studies with data (*n* = 278) (25, 27, 28) (Table 5). Amongst the 23 patients examined in the 18 case reports, 3 cases (13%) required mechanical ventilation (Table S6 and Figure S4). Antiviral agents (Oseltamivir, ritonavir, and lopinavir) were used in a high proportion of adult patient populations (84%, 95% CI: 74–90%) among four studies with data (*n* = 415). Corticosteroids use was 29% (CI: 18–42%) in these studies (Table 5).

**Figure 3:**
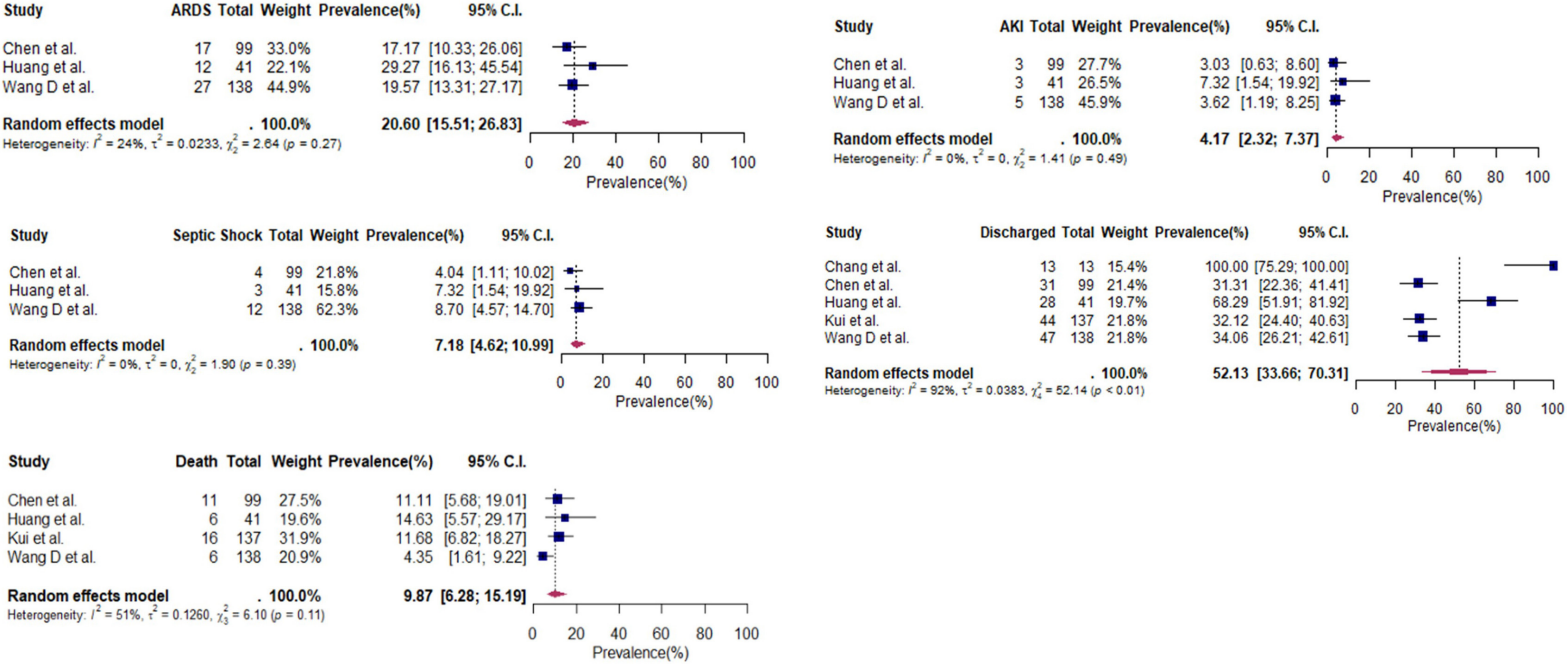
Forest plots for complications and outcomes (cross-sectional studies and case series).

For complications experienced during hospitalization, data was only provided by three studies using Wuhan hospital patients (*N* = 278). At the time of reporting, acute kidney injury (4%) and septic shock (7%) occurred in small proportion of patients (Table 6 and Figure 3). Case fatality rates amongst these studies was at 10% (95% CI: 6–15%) (Table 6). Conversely, no fatal cases were featured in the 23 patients from selected case reports (Table S7).

**Table 6:** Complications and outcomes of selected studies.

**References**	***N* (% with pneumonia)**	**ARDS**	**AKI**	**Septic Shock**	**Discharged**	**Death**
**CROSS-SECTIONAL STUDIES AND CASE SERIES**						
Chan et al. (30)	6 (100%)	–	–	–	–	–
Chang et al. (31)	13 (62%)				13 (100%)	–
Chen et al. (27)	99 (100%)	17 (17%)	3 (3%)	4 (4%)	31 (31%)	11 (11%)
Chung et al. (32)	21 (85%)	–	–	–	–	–
Huang et al. (25)	41 (100%)	12 (29%)	3 (7%)	3 (7%)	28 (68%)	6 (15%)
Kui et al. (26)	137 (100%)	–	–	–	44 (32.1%)	16 (11.7%)
Song et al. (33)	51 (100%)	–	–	–	–	–
Wang et al. (28)	138 (100%)	27 (20%)	5 (4%)	12 (9%)	47(34%)	6 (4%)
Wang et al. (34)	17 (Unspecified)	–	–	–	–	17 (100%)
杨涛/Yang et al. (35)	10 (100%)	–	–	–	–	–
马慧静/Ma et al. (29)^*^	22 (86%)	–	–	–	5 (23%)	–
	**Pooled prevalence (95% CI)**	**21% (16–27%)**	**4% (2–7%)**	**7% (5–11%)**	**52% (34–70%)**	**^∧^10% (6–15%)**
CASE REPORTS (WORLDWIDE)^**^						
Bastola et al. (Nepal) (24)	1 (Pneumonia)				Yes	
Holshue et al. (USA) (22)	1 (Pneumonia)	No	No	No	No	
Kim et al. (S. Korea) (23)	1 (Pneumonia)	–	–	–	No	
Phan et al. (Vietnam) (20)	1 (Pneumonia)	No	No	–	Yes	
Rothe et al. (Germany) (21)	1 (Mild infection)	–	–	–	Yes	–

* *Study 马慧静/Ma et al. not included in pooled figures as subjects are children*.

** *Case reports from patients in China found in Supplementary Material*.

∧ *Not including death case series from China*.

*ARDS, Acute Respiratory Distress Syndrome; AKI, Acute Kidney Injury*.

## Discussion

The novel pathogen SARS-CoV-2 is increasingly infecting more susceptible individuals, resulting in the spread of coronavirus disease (COVID-19) around the world (36). However, there is still limited knowledge in the key characteristics of populations-at-risk, including the clinical presentation and severity of patients during the early phase of this pandemic. Among several hypotheses about the disease, one postulation is that individuals infected by SARS-CoV-2 during the early phase of this pandemic had more severe outcomes (37).

As of 11th February, there was still a scarcity of literature published on the epidemiology and clinical characteristics of COVID-19 patients. Out of 10 selected studies with adult patients (all from China), subjects were primarily hospitalized COVID-19 cases with pneumonia (at least 508 out of 533 COVID-19 positive cases). These numbers are not representative of the disease spectrum as only patients with severe symptoms were more likely to seek medical attention at hospitals, with the Chinese CDC estimating that 81% of COVID-19 cases had no pneumonia in actuality (38).

The pooled median age of 56 among patients (95% CI: 49–57; 434 out of 451 adult patients with data had pneumonia) in our meta-analysis was consistent with understanding that older patients are more vulnerable to COVID-19 pneumonia (39). This was also reflected in the lower prevalence of symptoms at admission amongst COVID-19-infected children at Wuhan Children's Hospital (29). It has been proposed that older patients have weakened innate immunity accompanied by an over-reactive adaptive immune system induced by SARS-CoV-2, which leads to inflammatory responses like the “cytokine storm,” causing complications including pneumonitis and acute respiratory distress syndrome (ARDS) (40). Conversely, the innate immunity in children appears to block the viral invasion at the mucosal level, resulting in minimal to no symptoms, even as their adaptive immunity are relatively undeveloped (41). However, this requires further investigation.

This age distribution is similar to MERS-CoV which has been observed to affect children less compared to adults (42). A global study on the epidemiology of MERS-CoV in 2012–2013 reported that the median age of 161 infected patients was 50 years (range from 14 months to 94 years) (43). Conversely, SARS-CoV tends to infect younger individuals in China with a median age of 33 (44).

Amongst the 10 studies selected for meta-analysis, there was a 60% pooled prevalence of male patients. On the other hand, a February report by the WHO-China Joint Mission on COVID-19 found that 51.1% of 55,924 laboratory-confirmed infections were male. This discrepancy maybe due to selected studies' focus on pneumonia cases, as studies have shown that males tend to experience worse outcomes in COVID-19 infections compared to females (45). In contrast, WHO figures on MERS-CoV from affected countries worldwide showed that males made up 64% of cases (43). However, SARS-CoV has shown a different gender ratio. In China, 49% of the cases were female (46), while Singapore and Vietnam reported higher percentages of affected females (67.6 and 62.9%, respectively) (47, 48). The gender difference in these areas was attributed to the fact that hospital transmission of SARS occurred more in the latter two countries (44).

At least 45% of cases in our pooled patient population, which consisted predominantly of pneumonia cases, had existing comorbidities at admission. The most prevalent comorbidities were diabetes, hypertension, and cardiovascular disease. The WHO-China Joint Mission on COVID-19 also reported that Chinese patients with comorbidities had higher case fatality rates (13.2% with cardiovascular disease, 9.2% with diabetes, 8.4% with hypertension, 8.0% with chronic respiratory disease) (49). Hypertension, COPD and cardiovascular disease were also more common among ICU patients compared to non-ICU patients. Laboratory studies suggested this may be mediated by the Angiotensin-converting enzyme 2 (ACE2), a functional receptor for SARS-CoV-2 which the virus spike proteins bind to, and is highly expressed in the heart and lungs (50). This increases the likelihood of more severe complications, such as acute lung injury and acute myocarditis during COVID-19 infection among individuals with these existing comorbidities.

In the case of SARS, a review of studies across the world showed that pre-existing diabetes was a prognostic factor for worse outcomes (51). Conversely, the 2012–2013 global MERS-CoV study found that fatal MERS-CoV infections had a higher proportion of chronic kidney failure (20.8%) compared to recovered/asymptomatic cases (6.1%) (43).

From our pooled results, the common symptoms presented at admission were consistent with another study comprising of 1,099 COVID-19 patients (91.1% with pneumonia diagnosis) across 552 Chinese hospitals up till January 29th 2020 (52). Patients in Guan et al.'s study reported fever (43.8%), cough (67.8%), and fatigue (38.1%) at admission; in comparison this study's population had a pooled prevalence of 90, 58, and 50% for fever, cough, and fatigue, respectively. In Guan et al.' s study, vomiting (5%) and diarrhea (3.8%) were also less common (52). This finding was consistent in our pooled results (4% vomiting and 8% diarrhea). There are potential implications in active surveillance and triage if infected cases present with either cough only, fatigue only or diarrhea only. From our wide spectrum review of the clinical symptoms, sore throat or pharyngodynia was not a rare symptom at admission with pooled prevalence of 7% (95% CI: 1–15%). This observation is similar to other studies which reported 12.4 and 13.9% during presentation (53–55). This shows that sore throat should also be one of the clinical criteria taken into consideration during triage of suspected cases for further assessment. Olfactory and gustatory dysfunctions as a clinical presentation was reported among European, American, and Iranians (56, 57). However, these were not reported or observed among the Chinese patients during the early phase of the pandemic. This may be due to a few reasons. First, the differences could be due to a lack of awareness among the healthcare workers in the population to look out for such symptoms because these symptoms were also not known to be specific, resulting in lack of data among the Chinese patients. Second, the differences could be due to the fact that olfactory disorder may appear before the rest of the complaints as observed in 11.8% of cases (57). Third, this may be due to differences in genetic and physiological background between these populations and Asians. Lastly, the differences may be due to the different viral strains that circulated in these different regions (58).

With SARS, the most prominent symptoms on admission are cough, malaise, headache, and fever, according to a review on the global 2002–2003 epidemic (59). In a study comparing SARS with COVID-19, it was reported that symptoms for COVID-19 are similar to SARS (60). For MERS-CoV infections observed in 47 pneumonic patients from Saudi Arabia, common symptoms at presentation included fever (98%), cough (83%), shortness of breath (72%), and myalgia (32%). Gastrointestinal symptoms were also more frequent, including diarrhea (26%), vomiting (21%), and abdominal pain (17%) (61). The MERS coronavirus has been known to affect gastrointestinal tract (62).

This review's pooled prevalence of imaging features for at least 250 patients show that ground-glass opacity was at 59% and consolidation at 31%. This is consistent with one of the largest cross-sectional imaging studies of 1,014 suspected pneumonia patients in Wuhan, where ground-glass opacity (46%) and consolidation (50%) were main CT findings (63). Bilateral involvement amongst this group was also about 90% in pooled analysis which is similar to another study with 1,014 patients (63). In contrast, the hallmark imaging features of SARS tend to be unilateral at admission, before becoming bilateral with maximal lung involvement (64). On the other hand, the CT findings of MERS-CoV patients consist of more extensive ground-glass opacities than consolidation, with predominantly subpleural and basilar airspace changes (65).

In terms of COVID-19 treatment, there was heterogeneity across the different studies, especially with the use of invasive mechanical ventilation in Wuhan hospitals. In Huang et al.'s study, 15% of all COVID-19 patients admitted to ICU received mechanical ventilation, with 85% of this group experiencing ARDS during hospitalization (25); on the other hand, Wang et al. reported 47% of COVID-19 patients admitted to ICU received mechanical ventilation even as 61% of this group experienced ARDS (28). This may be due to a lack of mechanical ventilators as one review estimated that only 25% of COVID-19 fatalities in China were intubated and received mechanical ventilation (66). However, the selected studies in this review did not make reference to challenges in resource management (25, 28).

In selected cross-sectional studies with information on patients' outcomes as of last follow-up, the pooled case fatality rate (CFR) was 9.9%. This stands higher than China's CFR of 2.3% as of 11th February (67)—a result of our study population consisting predominantly of pneumonia cases. By comparison, the case fatality rate for MERS-CoV was 60%, much higher than that for COVID-19 (61).

One key limitation of this study was publication bias, as patients represented in this review are only a handful of patients that were reported. There were limited peer-reviewed studies, mainly case reports, case-series and cross-sectional studies that were published as of 11th February, resulting in a small study population that over-represented COVID-19 pneumonia cases. In order to achieve the most rigorous form of systematic review during the early phase of pandemic, only peer-reviewed articles but not preprints were included since peer-reviewing process is not yet a rate-limiting step. Moreover, heterogeneity of the studies (different hospital sites and patient composition) did not favor consistency in measurement of clinical variables, which may result in inaccurate meta-analysis. Hence, there is still a need to advocate for more and rapid sharing of these knowledge at the early phase of the pandemic, without just focusing on the severe outcomes to guide appropriate global responses and preparedness against COVID-19.

Furthermore, this review presents a cross-sectional view of COVID-19 patient characteristics during the early phase of the pandemic, and only 52% out of 450 patients with outcome reported were discharged at time of reporting. Hence outcomes of these patients, such as subsequent complications, ICU admission and deaths could have occurred after these studies have been published, and would introduce differential misclassification bias in the stratified analyses of ICU admission status.

Nevertheless, this systematic review will provide a basis for comparison of patient data between the early outbreak phase and the following months—including country-level comparisons. Case reports presented here also provide useful information on atypical COVID-infections; those found in our review include severe pneumonia in a child (68), a case of asymptomatic transmission (21), and the first imported cases in countries outside of China (22–24). In a nutshell, this knowledge will aid in formulating better detection strategy for surveillance and containment to minimize the spread of the SARS-CoV-2. As more literature becomes available, it would strengthen the next meta-analysis to provide a more accurate epidemiology and clinical characteristics of COVID-19 globally.

## Conclusions

Most eligible published literature was focused on severe outcomes at the early phase of the pandemic, which may not represent the true spectrum of epidemiological and clinical characteristics of COVID-19. The pooled analysis identified fever, cough, and myalgia as the most common symptoms at admission. Patients with shortness of breath at admission and pre-existing comorbidities are at higher risk of severe complications and fatality.

## Data Availability Statement

All datasets generated for this study are included in the article/Supplementary Material.

## Author Contributions

JP: conceptualization. PC, SS, JK, and JP: validation and investigation. HG, SS, and JK: formal analysis. PC, SS, and JK: data curation. JP, JK, PC, HG, and SS: writing—original draft preparation. JP and JK: writing—review and editing. HG and JK: visualization. All authors contributed to the article and approved the submitted version.

## Conflict of Interest

The authors declare that the research was conducted in the absence of any commercial or financial relationships that could be construed as a potential conflict of interest.
