# Supplementary material for: Epidemiological and Clinical Characteristics of Cases During the Early Phase of COVID-19 Pandemic: A Systematic Review and Meta-Analysis
Source: Front Med (Lausanne). 2020 Jun 11;7:295. doi: 10.3389/fmed.2020.00295 (PMC7300278; doi:10.3389/fmed.2020.00295)
Supplement: Supplementary file 1 [file Data_Sheet_1.docx]

**Supplementary Material**

# Table S1. PICOS eligibility criteria

| **Keywords** | “2019-nCoV”, “2019 novel coronavirus”, “nCoV”, “新型冠状病毒”, “新型肺炎” and “Wuhan pneumonia” |
| --- | --- |
| **Eligibility Criteria** | |
| Participants | Laboratory confirmed COVID-19 patients in any country |
| Exposure | Patient data (demographics, underlying comorbidities), clinical data (symptoms prior admission and at admission, chest imaging, treatment) |
| Comparator | None |
| Outcome | Microbiologically confirmed COVID-19 infection |
| Study design | Case reports, case series, correspondence, letters in peer reviewed journals |
| Language | English and Mandarin |
| Time- frame | All publications from 1^st^ January 2020 until 11^th^ February, 2020 |


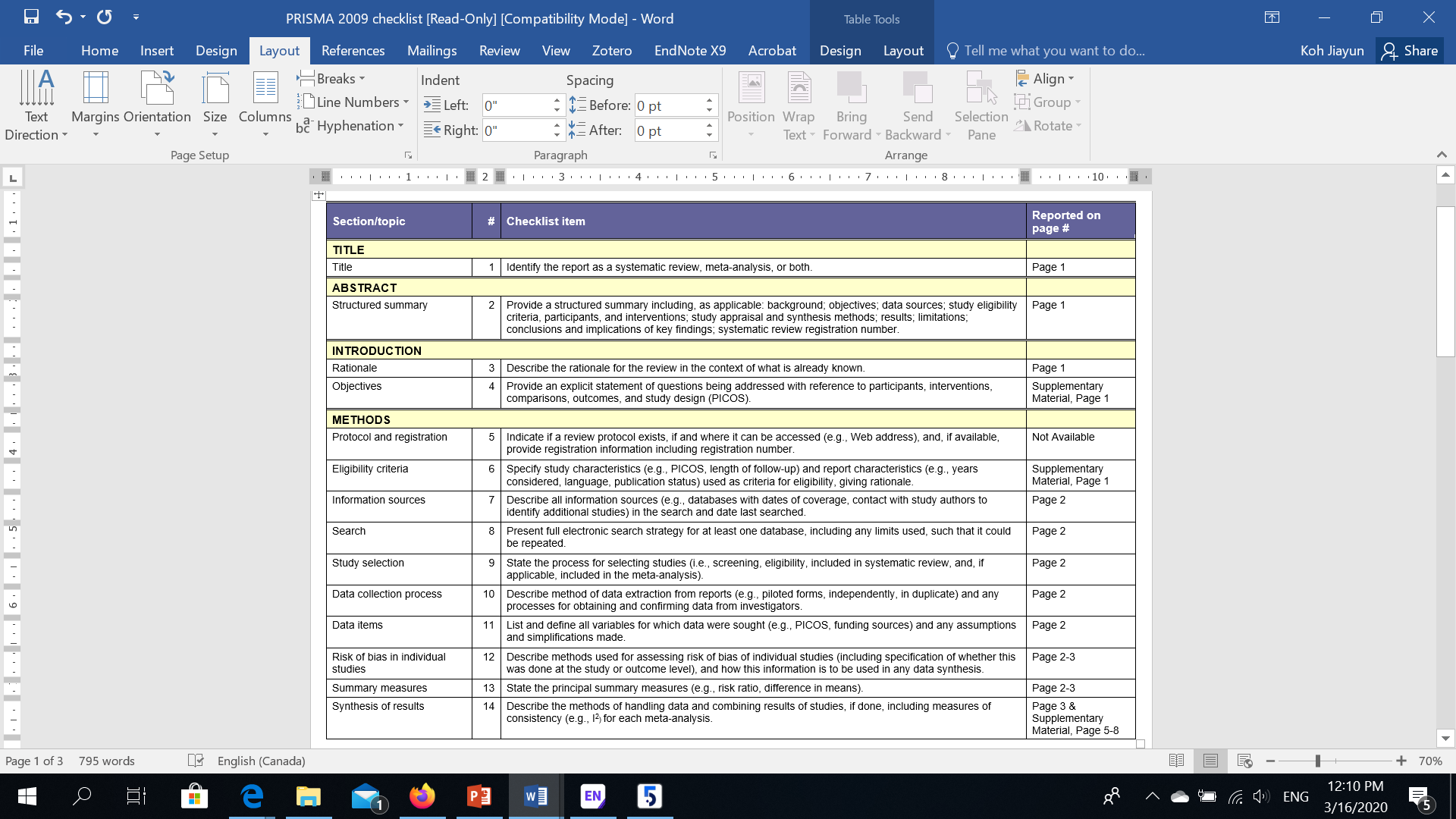
Figure S1. PRISMA Checklist


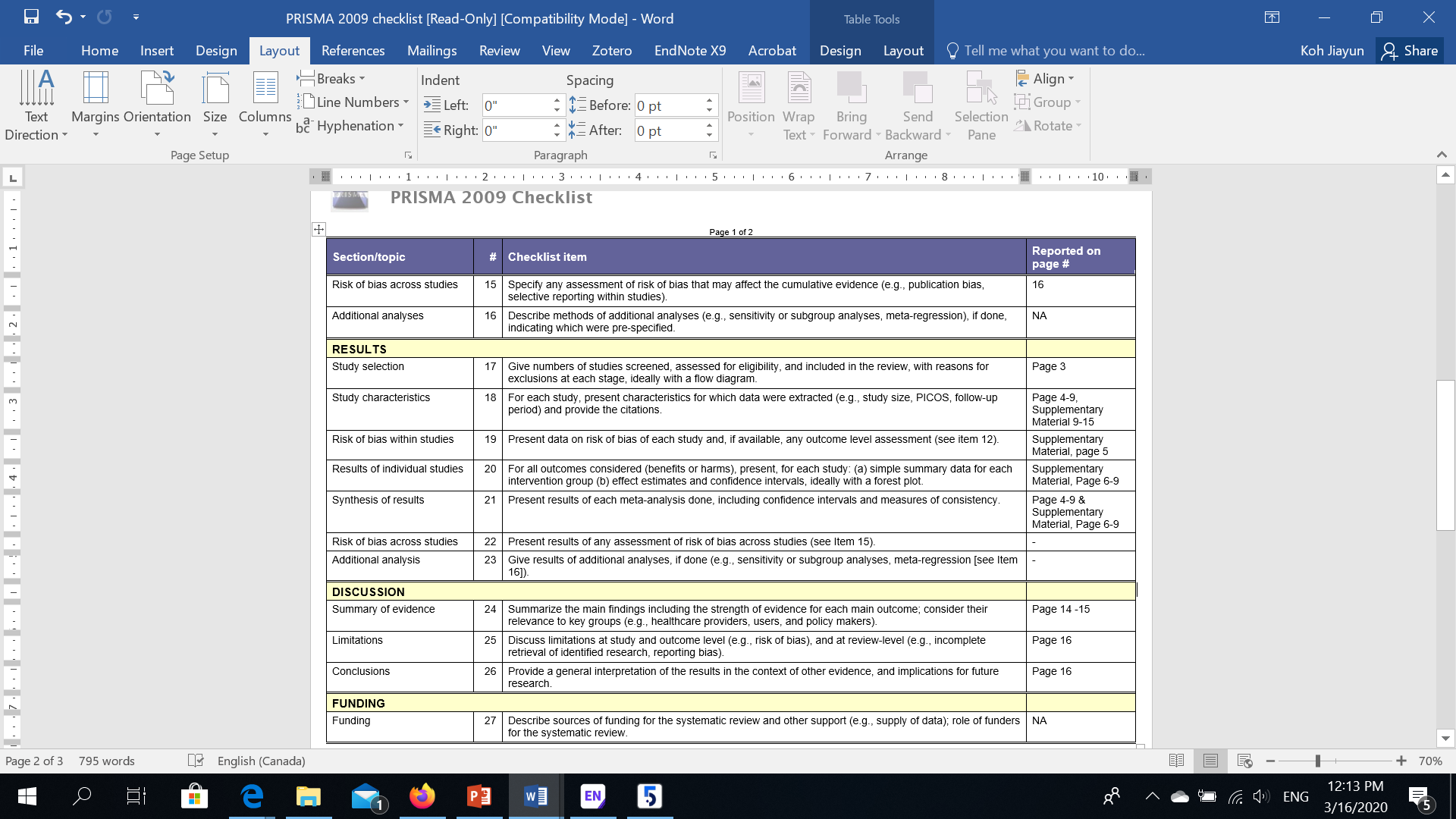


**Table S2.. Detailed Characteristics of selected studies**

| **Case series/Cross-sectional studies (All)** | **Author** | **Study Type** | **Publication Date** | **City, Country (Province)** | **Date of Admission** | **Site** |
| --- | --- | --- | --- | --- | --- | --- |
|  | Chan et al [[24](#_ENREF_24)] | Case series | 24-Jan | Shenzhen, Guangdong | 10-Jan-20 | The University of Hong Kong, Shenzhen Hospital |
|  | Chang et al. [[25](#_ENREF_25)] | Cross-sectional | 7-Feb | Beijing, China | 16-Jan-20 till 29-Jan-20 | 1.Beijing Tsinghua Changgung Hospital 2. Beijing Anzhen Hospital 3. Chinese PLA General Hospital |
|  | Chung et al. [[2](#_ENREF_2)] | Cross-sectional | 4-Feb | Qingdao, Zhuhai & Nanchang, China | 18-Jan-20 till 27-Jan-20 | 1. The First Affiliated Hospital of Nanchang University 2. The Affiliated Hospital of Qingdao University 3. The Fifth Affiliated Hospital, Sun Yat-sen University |
|  | Huang et al.[[19](#_ENREF_19)] | Cross-sectional | 24-Jan | Wuhan, China | 16-Dec-19 till 02-Jan-20 | Wuhan Jin Yin-tan hospital |
|  | Kui et al. [[20](#_ENREF_20)] | Cross-sectional | 5-Feb | Wuhan, Shiyan, Jingzhou, Yichang, Xiaogan/ China (Hubei) | 30-Dec-19 till 24-Jan-20 | 1.Tongji Hospital  2. Central Hospital of Wuhan  3. Taihe Hospital,  4. Jingzhou Central Hospital 5. The First People's Hospital of Jingzhou 6. The People's Hospital of Zhou 7. The Central Hospital of Xiaogan 8. The Sixth Hospital of Wuhan 9. Central Hospital of Enshi tujia & Miao Autonomous Prefecture |
|  | Chen et al. [[29](#_ENREF_29)] | Cross-sectional | 30-Jan | Wuhan, China | 01-Jan-20 till 20-Jan-20 | Wuhan Jin Yin-tan hospital |
|  | Song et al. [[26](#_ENREF_26)] | Cross-sectional | 6-Feb | Shanghai, China | 20-Jan-20 till 27-Jan-20 | Shanghai Public Health Clinical Centre |
|  | Wang D et al. [[22](#_ENREF_22)] | Cross-sectional | 7-Feb | Wuhan, China | 01-Jan-20 till 28-Jan-20 | Zhongnan Hospital of Wuhan University |
|  | Wang W et al. [[27](#_ENREF_27)] | Case series | 29-Jan | Across China including Wuhan | unknown | Across China, From National Health Commission Website |
|  | 杨涛/Yang et al.[[28](#_ENREF_28)] | Case series | 9 Feb | Shanghai, China | 01-Jan-20 & 03-Feb-20 | 1.Shanghai Jiao Tong Affiliated Sixth People's Hospital  2. Jinshan Branch Hospital |
|  | 马慧静/Ma et al. [[23](#_ENREF_23)] | Cross-sectional | 10 Feb | Wuhan, China | Diagnosis between 25-Jan-20 & 05-Feb-20 | Wuhan Children’s' Hospital |
| **Case Reports** | **Author** |  | **Publication Date** | **City, Country (Province)** | **Date of of Admission** | **Site** |
|  | Cai et al. [[6](#_ENREF_6)] | Case Report | 2-Feb | Shanghai, China | 19-Jan-20 | Children's Hospital of Fudan University, Shanghai branch |
|  | Fan Wu et al. [[7](#_ENREF_7)] | Case Report | 3-Feb | Wuhan, China | 26-Dec-19 | Wuhan Central Hospital |
|  | Fang et al. [[8](#_ENREF_8)] | Case Report | 7-Feb | Zhejiang, China | Unknown | Taizhou Hospital of Wenzhou Medical University |
|  | Lei et al. [[9](#_ENREF_9)] | Case Report | 31-Jan | Lanzhou, China | Unknown | First Hospital of Lanzhou University |
|  | Zhu et al. [[10](#_ENREF_10)] | Case Report | 24 Jan | Wuhan, China | 27-Dec-19 | Wuhan Jin Yin-tan hospital |
|  | Shi et al. [[11](#_ENREF_11)] | Case Report | 7-Feb | Wuhan, China | 1-Jan-20 | Union Hospital, Tongji Medical College |
|  | Zhang Y et al. [[12](#_ENREF_12)] | Case Report | 11 Feb | Haikou, China | 26-Jan-20 | Children's Hospital of Fudan University, Haikou, Xiaogan branch |
|  | Zhang Z et al. [[13](#_ENREF_13)] | Case Report | 7-Feb | Wuhan, China | 27-Dec-19 till 30-Dec-19 | Renmin Hospital of Wuhan University |
|  | 陈锋/Chen et al. [[30](#_ENREF_30)] | Case Report | 8 Feb | Wuhan, China | 27-Jan-20 | Wuhan's Childrens' Hospital (ICU) |
|  | 李进/Lee et al. [[31](#_ENREF_31)] | Case Report | 2 Feb | Yibin, Sichuan, China | 24-Jan-20 | Second Affiliated Hospital, Sichuan University Huaxian Medical Centre |
|  | 童松/Tong et al. [[32](#_ENREF_32)] | Case Report | 5 Feb | Wuhan, China | Unknown | Union Hospital, Tongji Medical College |
|  | 邓慧玲/Zheng et al. [[33](#_ENREF_33)] | Case Report | 7 Feb | Yan'an (Xi an), China | 30-Jan-20 till 31-Jan-20 | Xi an Childrens' Hospital |
|  | 庄思颖 /Zhuang et al. [[34](#_ENREF_34)] | Case Report | 11 Feb | Wuhan, China | 20-Jan-20 | Zhongnan Hospital of Wuhan University |
|  | Phan et al. [[14](#_ENREF_14)] | Case Report | 22-Jan | Ho Chi Minh, Vietnam | 22-Jan-20 | Cho Ray Hospital |
|  | Rothe et al. [[15](#_ENREF_15)] | Case Report | 30-Jan | Munich, Germany | 24-Jan-20 till 27-Jan-20 | Munich infectious disease unit |
|  | Holshue et al. [[16](#_ENREF_16)] | Case Report | 31-Jan | Washington, USA | 19-Jan-20 | Providence Regional Medical Centre |
|  | Kim et al. [[17](#_ENREF_17)] | Case Report | 2-Feb | Incheon, Korea | 19-Jan-20 | Seoul National University College of Medicine |
|  | Bastola et al. [[18](#_ENREF_18)] | Case Report | 10-Feb | Kathmandu, Nepal | 3-Jan-20 | Kathmandu hospital |


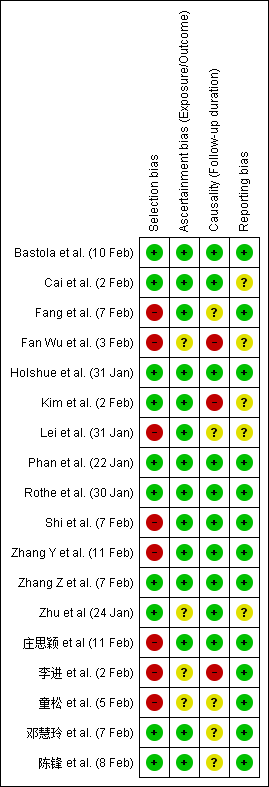
 Figure S2. Risk of Bias Summary for Case Series/Cross-sectional studies (Left) & Case Reports (right), based on Murad et al. Methodology Assessment tool


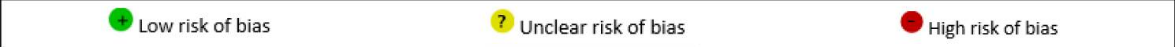

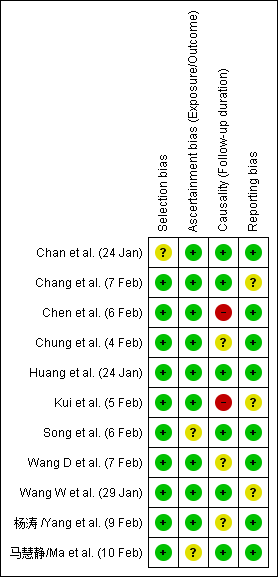
 Figure S3. Forest Plots for Pooled Prevalence of Epidemiological Characteristics & Comorbidities at Admission (Cross-sectional studies & case series)

For studies where there might be selection bias i.e. no selection criteria or reason for case selection— unclear or high risk was indicated under the selection domain in the bias assessment. The ascertainment domain, took into account the exposure/outcome ascertainment bias (one consideration was the presence of a consistent data collection tool for all subjects). The causality domain considered the follow-up time of subjects and if it was long enough for case classification to be reliable. The reporting domain assessed if the study reported sufficient details to allow practitioners to make useful inferences.


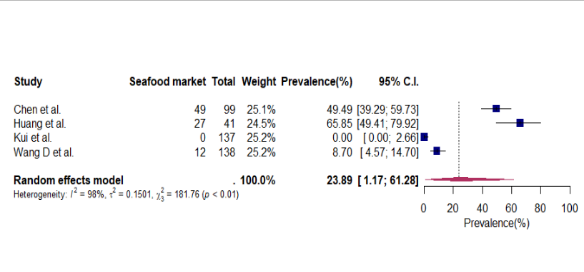

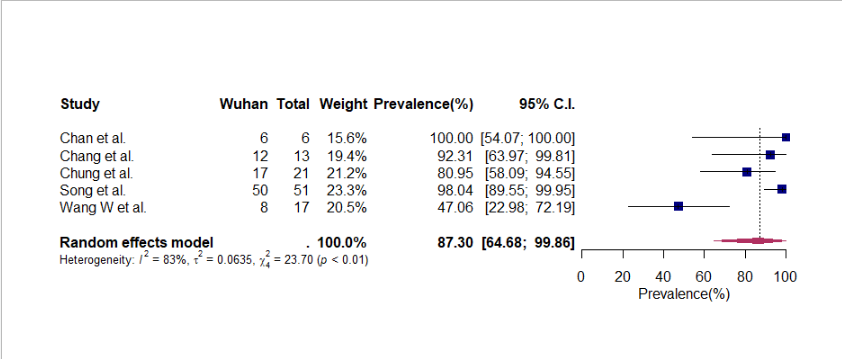

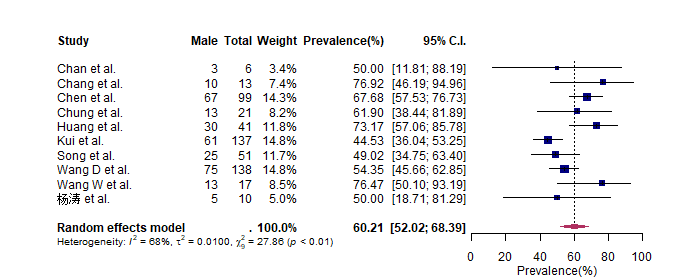


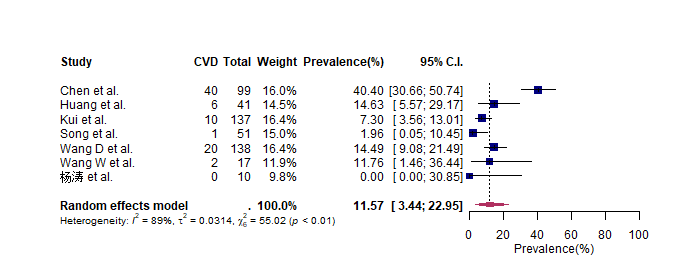

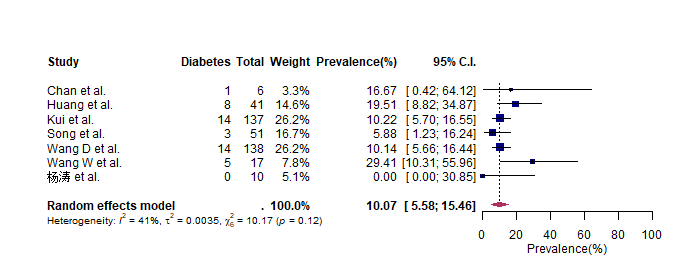

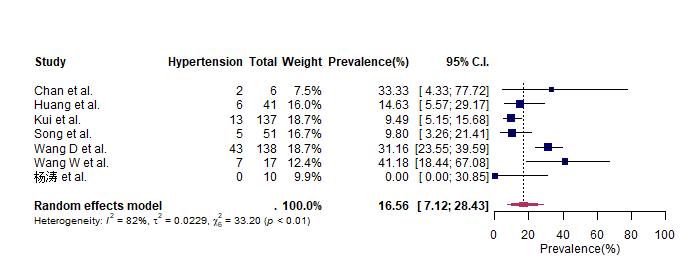


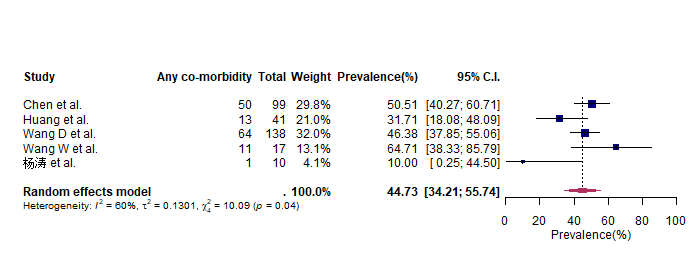

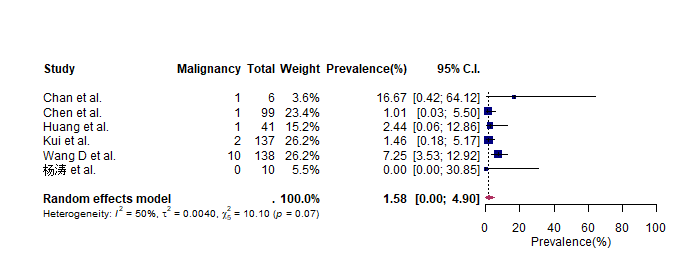

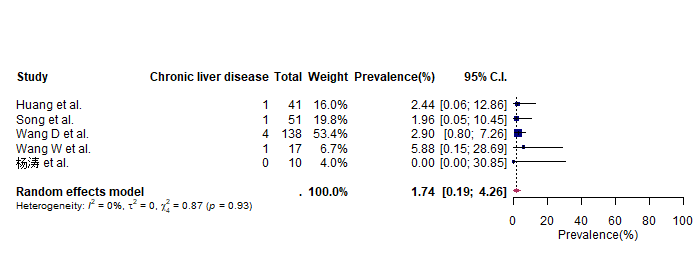

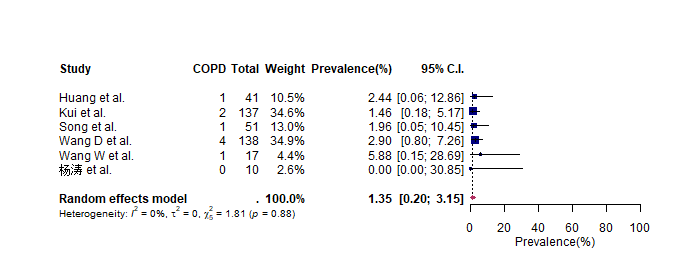


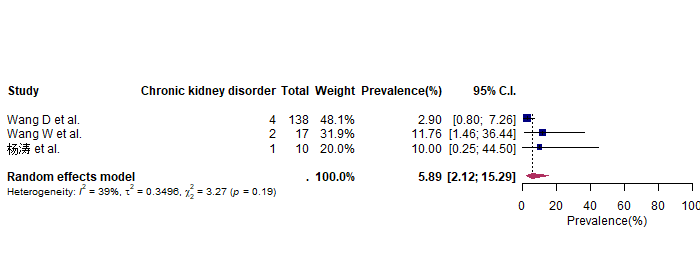


Figure S4. Forest Plots for Pooled Prevalence of Imaging results at admission & Treatment (Cross-sectional studies & case series)


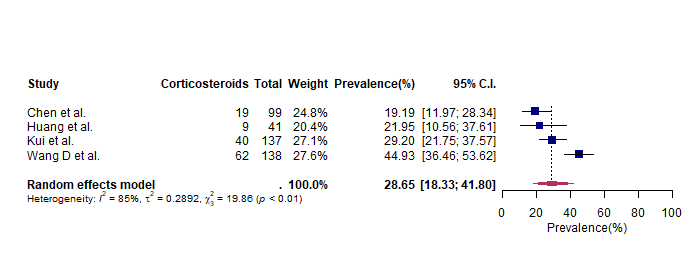

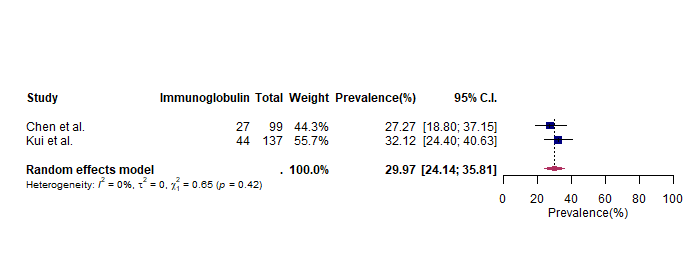

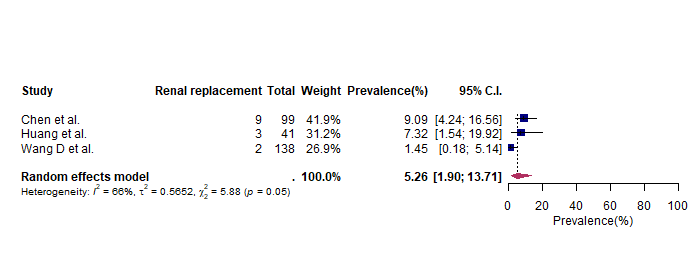

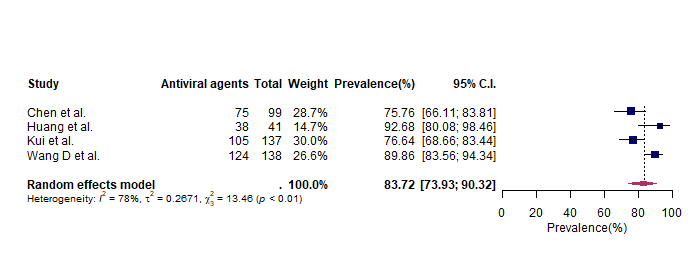

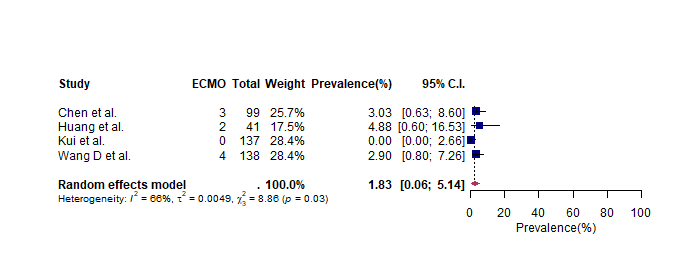

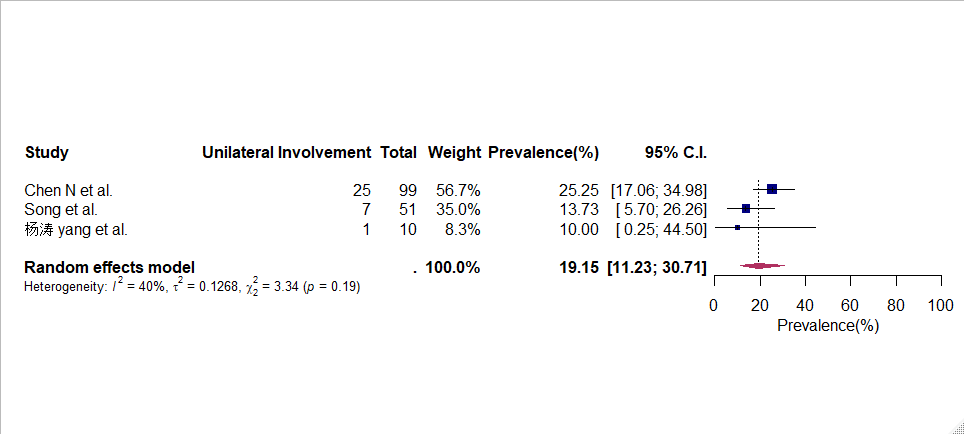

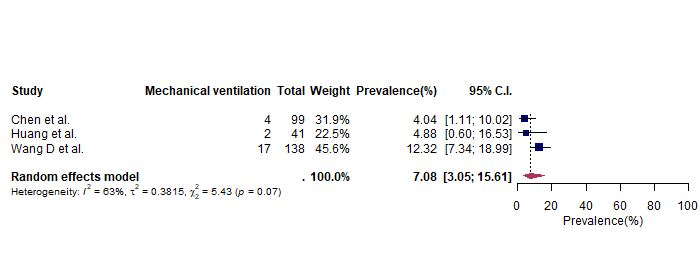

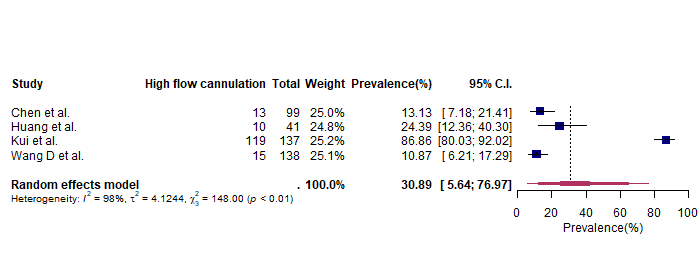

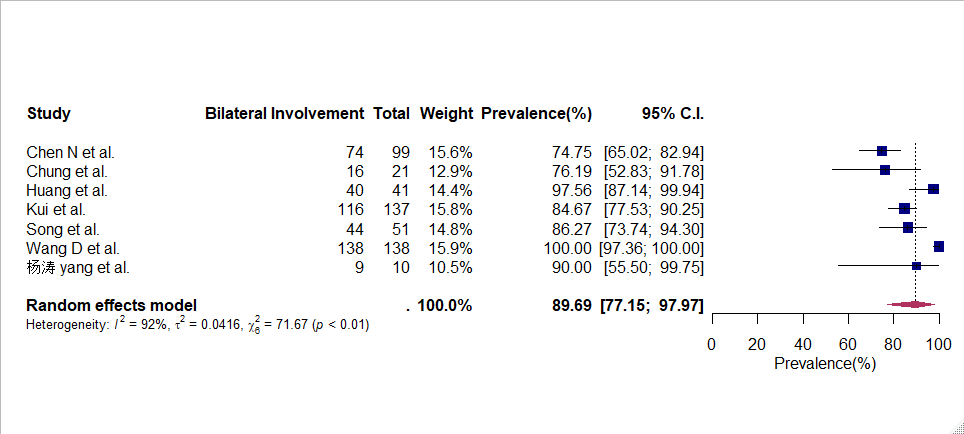

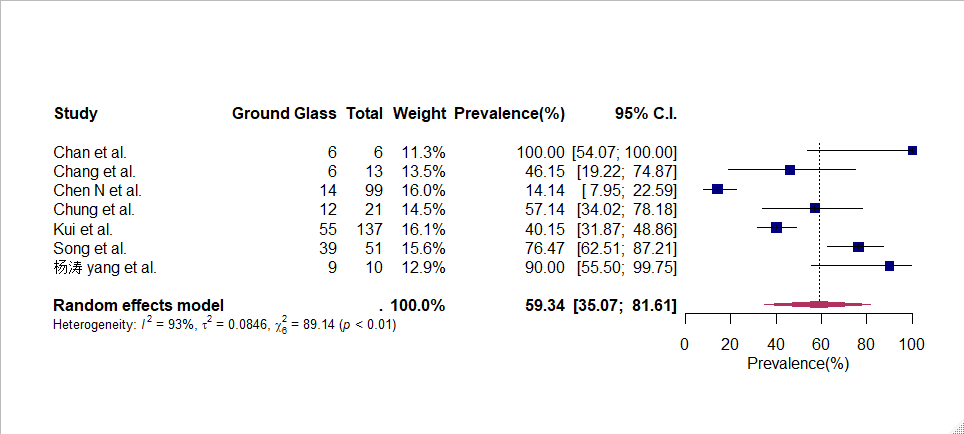

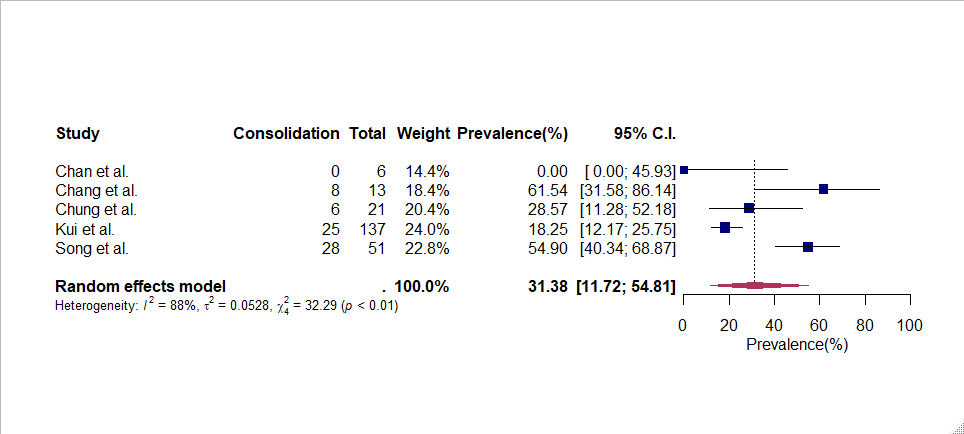


# Table S3. Patient Demographics & comorbidity for case reports

| **Author** | **No. of subjects** | **Case Classification** | **Age** | **Gender** | **Occupation** | **Epidemiological link with Wuhan** | **Duration from symptoms to medical event** | **Chronic conditions** |
| --- | --- | --- | --- | --- | --- | --- | --- | --- |
| Bastola et al. | 1 | Pneumonia | 32 | Male | Student at Wuhan University of Technology | Studying in Wuhan; No exposure seafood market | To discharge - 13 days | None |
| Cai et al. | 1 | Mild infection | 7 | Male | - | Visited family in Wuhan | To admission - 1 day | None |
| Fan Wu et al. | 1 | Pneumonia | 41 | Male | Vendor at Local indoor seafood market | Exposed to indoor seafood market | To hospitalisation - 6 days | Only mild lymphopenia and hypoxaemia |
| Fang et al. | 2 | Pneumonia | 45 | Female | - | Travel to Wuhan/ live in Wuhan | - | - |
|  |  | Pneumonia | 32 | Male | Businessman | Business visit to Wuhan | To clear CT - 8 days  To recovery - 10 days | - |
| Holshue et al. | 1 | Pneumonia | 35 | Male | - | Visited family in Wuhan | To admission - 4 days  To positive chest radiograph - 5 days To clear chest radiograph - 9 days To recovery - 12 days | Hypertriglyceridemia |
| Kim et al. | 1 | Pneumonia | 35 | Female | - | Travel history to Wuhan (live in Wuhan) | To resolution of fever - 11 days  To symptoms recovery - 14 days | Diabetes and Obese |
| Lei et al. | 1 | Pneumonia | 33 | Female | - | Worked in Wuhan and travelled to Lanzhou, China | To admission - 5 days | - |
| Phan et al. | 2 | Pneumonia | 65 | Male | - | Visited Wuhan  No exposure to wet market | To admission - 4 days  To stability - 13 days  To resolution of fever - 12 days | Diabetes, Hypertension, CVD, lung cancer Surgery - stent |
|  |  | Mild infection | 27 | Male | - | Met father who was infected | Incubation period - 3 days  To recovery - 3 days | None |
| Rothe et al. | 1 | Mild infection | 33 | Male | Businessman | Exposed by colleague from Shanghai *(asymptomatic at the point of time)* | To recovery - 4 days | None |
| Shi et al. | 1 | Pneumonia | 42 | Male | - | *Not applicable*  *(Wuhan hospital)* | To recovery - 31 days | - |
| Zhang Y et al. | 1 | Pneumonia | 3 Months 19 days | Female | - | Wuhan Citizen | To admission - 1 day  To discharge – 10 days | - |
| Zhang Z et al. | 2 | Pneumonia | 38 | Male | - | From Wuhan | To admission - 7 days  To stability - 14 days | Allergy to a Chinese traditional medicine |
|  |  | Pneumonia | 38 | Female | - | From Wuhan | To admission- 1 day | None |
| Zhu et al. | 2 | Pneumonia | 49 | Female | Retailer at seafood market | Exposed to the seafood market | To admission - 4 days  To discharge - 24 days | None |
|  |  | Critical pneumonia | 61 | Male | - | Frequent visitor to the seafood wholesale market | To admission - 7 days  To death - 20 days | - |
| 童松 et al. | 1 | Mild infection | 64 | Female | - | - | - | Hypertension, CVD |
| 陈锋 et al. | 1 | Critical pneumonia | 1 year  1 month | Male |  | From Wuhan | From admission to PCR 8 days From symptoms to PCR 14 days | None |
| 庄思颖 et al. | 1 | Pneumonia | 33 | Female | Tutor | From Wuhan; No exposure to Huanan seafood. | - | Pregnant, hepatic dysfunction |
| 李进 et al. | 1 | Pneumonia | 33 | Female | - | Visited Wuhan | To PCR confirmation - 6 days | - |
| 邓慧玲 et al. | 2 | Mild infection | 3 years 10 months | Female | - | Wuhan citizen | To admission - 1 day | None |
|  |  | Mild infection | 13 | Male | - | Live in Wuhan | To admission - 3 days | None |

# Table S4. Symptoms before admission & first symptoms to appear for case series & case reports

| **Author** | **Publication Date** | **Symptoms prior to seeking medical care (First symptom to appear underlined)** |
| --- | --- | --- |
| Bastola et al. | 10-Feb | Cough |
| Cai et al. | 2-Feb | Fever, cough, & runny nose (1 day) |
| Chan et al | 24-Jan | **Patient 1**: Fever, cough & fatigue  **Patient 2**: Fever, fatigue & cough  **Patient 3**: Fever, diarrhoea & pleuritic chest pain  **Patient 4**: Fever, diarrhoea, cough & runny nose  **Patient 5**: Back pain, fatigue, fever & dry cough |
| Fan Wu et al. | 3-Feb | Fever, chest tightness, unproductive cough, pain & weakness (7 days) |
| Holshue et al. | 31-Jan | Fever (3 days) & cough (4 days); nausea (2 days), vomiting, Fatigue (3 days) |
| Kim et al. | 2-Feb | Fever, chill, and myalgia for 1 day |
| Lei et al. | 31-Jan | Fever & Cough (5 days) |
| Phan et al. | 22-Jan | **Patient 1:** fever & fatigue (4 days) (father)  **Patient 2:** vomiting and loose stools, dry cough and fever (2 days) (son) |
| Rothe et al. | 30-Jan | Sore throat, chills, myalgia, productive cough & fever |
| Shi et al. | 7-Feb | Cough, fatigue & high-grade fever (1 week) |
| Zhang Y et al. | 11 Feb | Fever (1 day) |
| Zhang Z et al. | 7-Feb | **Patient 1**: Fever (1 week) & dyspnea (1 day)  **Patient 2**: Fever, cough & vomiting (1 day) |
| Zhu et al. | 24-Jan | **Patient 1** Fever, cough, chest discomfort (4 days)  **Patient 2**: Fever, cough & respiratory distress (7 days) |
| 李进 / Lee et al. | 2 Feb | Fever & fatigue (1 day) |
| 邓慧玲/ Zheng et al. | 7 Feb | **Patient 1:** Fever  **Patient 2:** Sore throat |

# Table S5. Symptoms at admission for case reports

| **Author** | **Case Classification** | **Fever (%)** | **Cough (%)** | **Sputum (%)** | **Sore throat (%)** | **Shortness of breath (%)** | **Vomiting (%)** | **Myalgia (%)** | **Malaise/Fatigue (%)** | **Rhinorrhoea (%)** | **Headache (%)** | **Diarrhoea (%)** | **Chest pain (%)** |
| --- | --- | --- | --- | --- | --- | --- | --- | --- | --- | --- | --- | --- | --- |
| Bastola et al. | Pneumonia | Yes | Yes | - | - | Yes | - | - | - | - | - | - | - |
| Cai et al. | Pneumonia | Yes | Yes |  | - |  | - | - | - | Yes | - | - |  |
| Fan Wu et al. | Pneumonia | Yes | Yes | Yes | - | Yes | - | - | - | - | - | - | Yes |
| Fang et al. | Pneumonia | Yes | Yes | - | - | - | - | - | - | - | - | - | - |
| Holshue et al. | Pneumonia | Yes | Yes | - |  | - | Yes | - | Yes | - | - | - | - |
| Kim et al. | Pneumonia | Yes |  | - | Yes | - | - | Yes | - | - | - | - | - |
| Lei et al. | Pneumonia | Yes | Yes | - | - | - | - | - | - | - | - | - | - |
| Phan et al. | Pneumonia | Yes | - | - | - | Yes | - | - | Yes | - | - | - | - |
|  | Mild infection | Yes | Yes | - | - | - | Yes | - | - | - | - | Yes | - |
| Rothe et al. | Mild infection | Yes | Yes | Yes | Yes | - | - | Yes | Yes | - | - | - | - |
| Shi et al. | Mild infection | Yes | Yes | - | - | - | - | - | Yes | - | - | - | - |
| Zhang Y et al. | Pneumonia | Yes | **-** | - | - | - | - | - | - | - | - | - | - |
| Zhang Z et al. | Pneumonia | Yes | Yes | Yes | - | Yes | - | - | - | - | - | - | Yes |
|  | Pneumonia | Yes | Yes | - | - | - | Yes | - | - | - | - | - |  |
| Zhu et al. | Pneumonia | Yes | Yes | - | - | - | - | - | - | - | - | - |  |
|  | Pneumonia | Yes | Yes | - | - | - | - | - | - | - | - | - | Yes |
| 童松 et al. | Mild infection | Yes | - | - | - | - | - | - | - | - | - | - | - |
| 陈锋 et al. | Pneumonia | Yes | - | - | - | Yes | Yes | - | Yes | - | - | Yes | - |
| 庄思颖 et al. | Pneumonia | Yes | Yes | - | - | - | - | - | Yes | - | - | - | - |
| 李进 / Lee et al. | Pneumonia | Yes | - | - | - | - | - | - | Yes | - | - | - | - |
| 邓慧玲/ Zheng et al. | Mild infection | Yes | - | - | - | - | - | - | No | - | - | - | - |
|  | Mild infection |  |  | - | Yes | - | - | - |  | - | - | - | - |

# Table S6. Chest Imaging at admission & Treatment in case reports

| **Author** | **Case Classification** | **Ground Glass** | **Consolidation** | **Infiltrate** | **Unilateral**  **Involvement** | **Bilateral**  **Involvement** | **Mechanical ventilation** | **High flow cannulation** | **ECMO** | **Antiviral agents** | **Antibiotic therapy** | **Corticosteroids** | **Immunoglobulin** |
| --- | --- | --- | --- | --- | --- | --- | --- | --- | --- | --- | --- | --- | --- |
| Bastola et al. | Pneumonia | - | - | Yes | - | - | No | - | - | - | Yes | - | - |
| Cai et al. | Pneumonia | - | - | - | - | - | No | - | - | - | - | - | - |
| Fan Wu et al. | Pneumonia | Yes | Yes | - | No | Yes | Yes | - | - | Yes | Yes | No | - |
| Fang et al. | Pneumonia | - | Yes |  | No | Yes | No | - | - | Yes | - | - | - |
|  | Pneumonia | - | Yes | - | No | Yes | No | - | - | Yes | - | - | - |
| Holshue et al. | Pneumonia | - | - | - | - | - | No | Yes | - | Yes | - | - | - |
| Kim et al. | Pneumonia | Ye s | Yes | Yes | - | - | No | Yes | - | Yes | - | - | - |
| Lei et al. | Pneumonia | Yes | - | - | No | Yes | No | - | - | - | - | - | - |
| Phan et al. | Pneumonia | - | Yes | - | - | - | No | Yes | - | Yes | Yes | - | - |
|  | Mild infection | - | - | - | - | - | No | - | - | - | - | - | - |
| Rothe et al. | Mild infection | - | - | - | - | - | No | - | - | - | - | - | - |
| Shi et al. | Mild infection | Yes | Yes | - | - | - | No | - | - | Yes | Yes | Yes | - |
| Zhang Y et al. | Pneumonia | - | - | - | - | - | No | - | - | Yes | - | - | - |
| Zhang Z et al. | Pneumonia | Yes | - | - | No | Yes | No | - | - | Yes | Yes | Yes | Yes |
|  | Pneumonia | Yes | - | - | No | Yes | No | - | - | Yes | Yes | Yes | No |
| Zhu et al. | Pneumonia | - | - | - | - | - | No | - | - | - | - | - | - |
|  | Pneumonia | - | - | - | - | Yes | Yes | - | - | - | - | - | - |
| 童松 et al. | Pneumonia | No | No | No | - | - | No | - | - | Yes | Yes | - | Yes |
| 陈锋 et al. | Pneumonia | Yes | Yes | - | No | Yes | Yes | - | - | - | - | - | - |
| 庄思颖 et al. | Pneumonia | Yes | Yes | - | No | Yes | No |  |  | Yes |  | - | - |
| 李进 et al. | Pneumonia | Yes | - | - | No | Yes | - | - | - | - | - | - | - |
| 邓慧玲 et al. | Mild infection | No | No | No | No | No | No | No | No | Yes | - | - | - |
|  | Mild infection | No | No | No | No | No | No | No | No | Yes | - | - | - |

**Table S7. Complications & Outcomes of case reports**

| **Author** | **Classification** | **ARDS** | **AKI** | **Septic Shock** | **Hospitalised** | **Discharged** | **Death** |
| --- | --- | --- | --- | --- | --- | --- | --- |
| Bastola et al. | Pneumonia | - | - | - | No | Yes | No |
| Cai et al. | Pneumonia | - | - | - | - | Yes | No |
| Fan Wu et al. | Pneumonia | Yes | - | - | Yes | - | No |
| Fang et al. | Pneumonia | - | - | - | - | Yes | No |
|  | Pneumonia | - | - | - | Yes |  | No |
| Holshue et al. | Pneumonia | No | No | No | Yes | No | No |
| Kim et al. | Pneumonia | - | - | - | Yes | No | No |
| Lei et al. | Pneumonia | - | - | - | Yes | No | No |
| Phan et al. | Pneumonia | No | No | - | No | Yes | No |
|  | Mild infection | No | No | No | No | Yes | No |
| Shi et al. | Mild infection | No | No | No | No | Yes | No |
| Rothe et al. | Mild infection | No | No | No | Yes |  | No |
| Shi et al. | Pneumonia | - | - | - |  | Yes | No |
| Zhang Y et al. | Pneumonia | No | No | No | No | Yes | No |
| Zhang Z et al. | Pneumonia | No | No | No | No | Yes | No |
|  | Pneumonia | No | No | No | No | Yes | No |
| Zhu et al. | Pneumonia | Yes | No | No | No | Yes | No |
|  | Pneumonia | No | No | No | No | Yes | No |
| 陈锋 et al. | Pneumonia | Yes | Yes | Yes | Yes | No | No |
| 庄思颖 et al. | Unknown | No | No | No | Yes | Yes | No |
| 童松 et al. | Pneumonia | No | No | No | Yes | No | No |
| 李进 et al. | Pneumonia | - | - | - | Yes | No | No |
| 邓慧玲 et al. | Mild infection | No | No | No | Yes | No | No |
|  | Mild infection | No | No | No | Yes | No | No |
